# Evolution and Expression of Paxillin Genes in Teleost Fish

**DOI:** 10.1371/journal.pone.0165266

**Published:** 2016-11-02

**Authors:** Andrew E. Jacob, Christopher E. Turner, Jeffrey D. Amack

**Affiliations:** Department of Cell and Developmental Biology, State University of New York, Upstate Medical University, Syracuse, New York, 13210, United States of America; Institut Albert Bonniot-INSERMU823, FRANCE

## Abstract

**Background:**

Paxillin family proteins regulate intracellular signaling downstream of extracellular matrix adhesion. Tissue expression patterns and cellular functions of Paxillin proteins during embryo development remain poorly understood. Additionally, the evolution of this gene family has not been thoroughly investigated.

**Results:**

This report characterizes the evolution and expression of a novel Paxillin gene, called Paxillin-b, in Teleosts. Alignments indicate that Teleost Paxillin-a and Paxillin-b proteins are highly homologous to each other and to human Paxillin. Phylogenetic and synteny analyses suggest that these genes originated from the duplication of an ancestral Paxillin gene that was in a common ancestor of Teleosts and Tetrapods. Analysis of the spatiotemporal expression profiles of Paxillin-a and Paxillin-b using zebrafish revealed both overlapping and distinct domains for Paxillin-a and Paxillin-b during embryo development. Localization of zebrafish Paxillin orthologs expressed in mammalian cells demonstrated that both proteins localize to focal adhesions, similar to mammalian Paxillin. This suggests these proteins regulate adhesion-dependent processes in their endogenous tissues.

**Conclusion:**

Paxillin-a and Paxillin-b were generated by duplication in Teleosts. These genes likely play similar roles as Paxillin genes in other organisms. This work provides a framework for functional investigation of Paxillin family members during development using the zebrafish as an in vivo model system.

## Introduction

**Fig 1:**
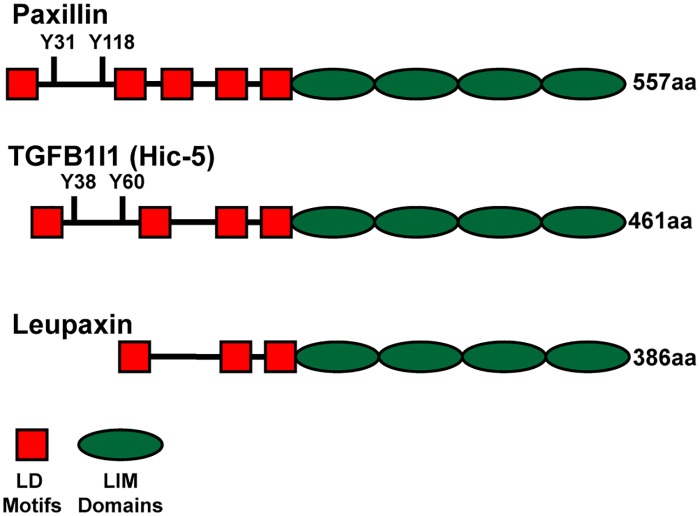
Human Paxillin family members. A schematic of the domain organization and length (in amino acids; aa) of the three Paxillin family proteins in humans. LD motifs (red), LIM domains (green) and N-terminal tyrosine (Y) phosphorylation sites are represented.

Cellular adhesion to the surrounding extracellular matrix (ECM) regulates many processes during tissue morphogenesis and animal development. Integrin adhesion to the ECM results in the assembly of intracellular complexes that regulate downstream signaling cascades and cytoskeletal rearrangements [1]. One class of proteins found in Integrin-adhesion complexes is the Paxillin family [2]. The human Paxillin family has three members, Paxillin, TGFβ1I1 (also known as Hic-5), and Leupaxin, which have a similar protein domain structure (Fig 1). Members of this family possess four C-terminal LIM domains, which are required for their localization to Integrin-adhesion complexes [3–5]. Additionally, amino-terminal features such as LD motifs and tyrosine phosphorylation sites are conserved between all family members [6]. Despite their similar structures, these proteins have been demonstrated to have both unique and complementary functions depending on cell type and context [7, 8].

Roles for Paxillin proteins during embryonic development have been identified using animal models, but their spatiotemporal expression patterns and mechanistic functions during development are just starting to be elucidated. Gain and loss-of-function experiments in *D*. *melanogaster* [9, 10] and *C*. *elegans* [11] embryos have identified roles for Paxillin orthologs in cytoskeletal organization required for morphogenesis and function of epithelia and muscles. In *X*. *laevis* embryos, Paxillin localizes to sites of muscle attachment [12, 13] and gain-of-function experiments suggest that an ortholog of TGFβ1I1 inhibits Wnt signaling [14]. In the early mouse embryo, Paxillin is primarily expressed in extraembryonic and mesoderm-derived tissues, as well as in migrating neural crest cells [15]. However, Paxillin knockout mice die at embryonic day 9.5 due to cardiovascular defects [15], which has limited developmental studies. TGFβ1I1 is mainly expressed in contractile cell types such as vascular smooth muscle and myoepithelial cells [16], but TGFβ1I1 knockout mice do not show developmental defects [17]. In the zebrafish (*D*. *rerio*) embryo, Paxillin proteins have been reported to localize at developing somite boundaries and vertical myosepta of the trunk [18]. These findings indicate Paxillin family members have both common and unique functions during embryonic development, but how this diversity emerged over evolutionary time remains unclear.

Three Paxillin family genes have been identified in mammals, while only one has been identified each in fungi, protozoans, and invertebrates [11, 19–21]. To investigate the evolutionary divergence of the Paxillin family, we identified orthologous genes in a wide array of taxa. Through this search we found a fourth Paxillin family member gene in Teleost fish, herein referred to as Paxillin-b (*pxnb*). Phylogenetic analysis using multiple protein alignment and interspecies synteny relationships suggested that Teleost Paxillin-a (*pxna*) and *pxnb* arose from a duplication of the ancestral Paxillin gene after Teleost divergence from other vertebrates. Using the zebrafish as a model Teleost, spatial and temporal expression of the duplicated Paxillin genes was determined during embryonic development. It was found that these genes have both overlapping and distinct expression patterns in the embryo. Interestingly, subcellular distributions of each protein were similar when exogenously expressed in cultured mammalian cells, suggesting conserved functions between these proteins and mammalian Paxillin. These results establish zebrafish as a useful model for investigating the functional roles of Paxillin family member genes during vertebrate development.

## Results and Discussion

### Identification of a novel Paxillin gene in Teleost fish

**Fig 2:**
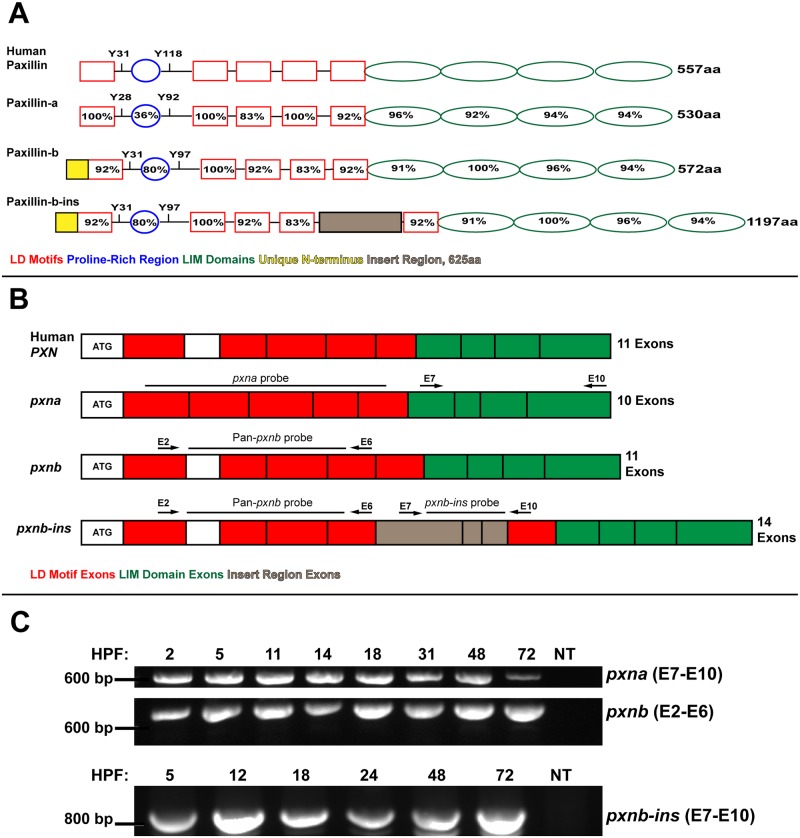
Alignments and expression of zebrafish Paxillin family members. (**A**) Schematics of zebrafish Paxillin-a (Pxna) and Paxillin-b (Pxnb) protein isoforms aligned with human Paxillin. The percentage of amino acids that are identical to human Paxillin is indicated for LD motifs (red), LIM domains (green), and the proline-rich region (blue). The Pxnb-ins isoform is predicted to contain a unique 625 amino acid insert region (brown) between LD4 and LD5. (**B**) Exon organization of zebrafish *pxna*, *pxnb* and *pxnb-ins* transcripts aligned with human *PXN*. Exons coding for LD motifs and LIM domains are red or green respectively. Alternatively spliced exons coding for the insert region of *pxn-ins* are colored in brown. Arrows depict primer sets used for RT-PCR and solid lines indicate positions of RNA *in situ* hybridization probes. (**C**) RT-PCR analysis of multiple stages of zebrafish development (hours post-fertilization; hpf) using primers to amplify *pxna*, *pxnb* or specifically *pxnb-ins* transcripts. NT = negative control.

A screen for orthologs of mammalian Paxillin in zebrafish was carried out using BLASTP search with full-length human Paxillin protein sequence as a query. Interestingly, searches against the zebrafish genome release version 9 [22], herein referred to as Zv9, identified a Leupaxin-like gene with significant homology within LIM domains and two other genes with significant similarity to the full-length query sequence. The first gene, Paxillin-a (*pxna*; Ensembl ID: ENSDART00000126598), is located on zebrafish chromosome 5 and is the zebrafish Paxillin that has been previously described [18, 23]. The second gene, Paxillin-b (*pxnb*, Ensembl ID: ENSDARG00000060766), was annotated as a predicted transcript encoded on chromosome 8. Zebrafish Pxna and the novel predicted Pxnb protein sequences share considerable homology with each other and with the human Paxillin protein (Fig 2A). Each has five N-terminal LD motifs and four C-terminal LIM domains, which are common characteristics of vertebrate Paxillin orthologs [24]. Additionally, each zebrafish protein has conserved tyrosine residues that correspond to Y31 and Y118 in human Paxillin (Fig 2A), which are kinase substrates in mammalian cells [25]. Interestingly the proline-rich region of human Paxillin, another site for protein-protein interactions [26], was more closely conserved in zebrafish Pxnb as compared to Pxna (Fig 2A). The predicted exon 1 of *pxnb* encodes a 47 amino acid sequence N-terminal to the first LD motif, which is not conserved in *pxna* or human Paxillin (Fig 2A and 2B). Also exclusive to the *pxnb* gene in Zv9 are three exons (E7-9) coding for a novel 625 amino acid insert region between the LD4 and LD5 motifs (Fig 2A and 2B). Herein, the isoform with this insert region will be called *pxnb-ins*. RT-PCR experiments using primer pairs specific for *pxna*, *pxnb* or *pxnb-ins* detected each transcript at multiple stages of zebrafish development (Fig 2C).

### Evolution of the Paxillin gene family

**Fig 3:**
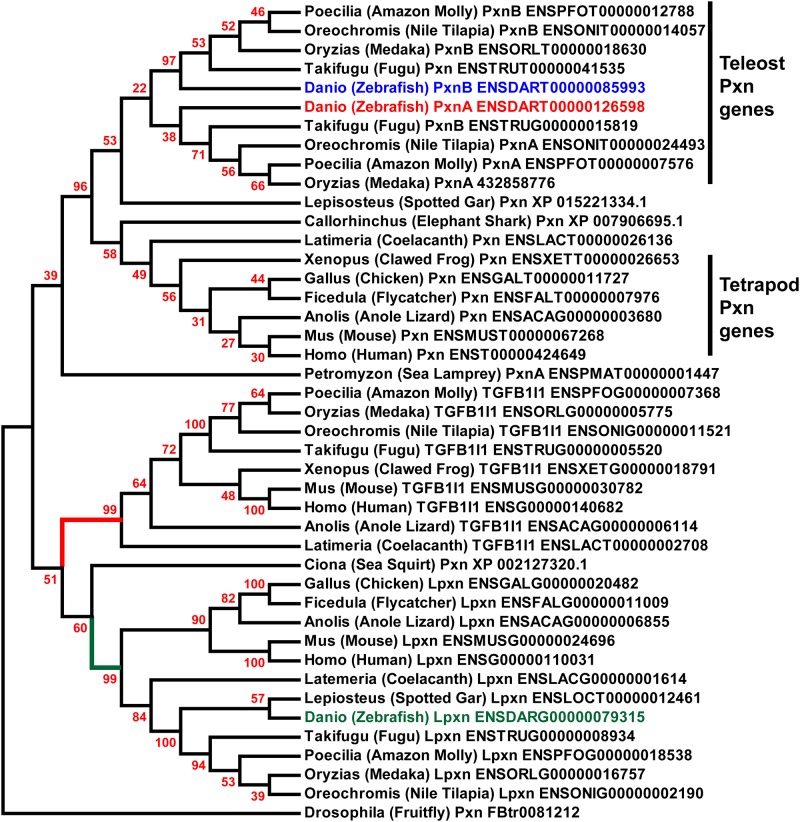
Phylogeny of Paxillin family genes. A maximum-likelihood phylogenetic tree of Paxillin gene family evolution. Nodes are labeled with percent support from 1000 bootstrap replicates. TGFβ1I1 and Leupaxin branches are labeled in red and green respectively. The presence of *pxna* and *pxnb* genes is restricted to Teleosts.

Identification of a *leupaxin* (*lpxn)* gene and two *pxn* genes, but no *tgfb1i1*gene, in the zebrafish was surprising given that Mammals have one copy of each Paxillin family member. To explore the divergence of the Paxillin family in a broader context, we identified Paxillin family genes (Table 1) from a wide array of taxa using zebrafish Pxnb as a protein BLAST query in selected species genomic databases. Multiple protein sequence alignments and generation of a phylogenetic tree revealed interesting insights into the evolution of the Paxillin family. Similar to the case in zebrafish, all Teleost fish assessed had two *pxn* genes (Fig 3). This observation is consistent with a whole-genome duplication event in the common ancestor of all Teleost fish [27]. However, other Teleost fish were found to have only single *lpxn* and *tgfb1i1* genes. These findings suggest that duplicated *pxn* genes have been retained in Teleosts, whereas duplicated *lpxn* and *tgfb1i1* genes were eliminated prior to Teleost species radiation and the *tgfb1i1* gene was lost entirely in the zebrafish lineage. Surprisingly though, we found that the N-terminal LD motifs of zebrafish Lpxn were highly conserved with those found in Tgfb1i1 of other species. Thus, we speculate that in zebrafish, *lpxn* substitutes for the role of *tgfb1i1*.

**Table 1:** Paxillin Family Members Identified.

Scientific Name	Common Name	Taxon	Genome Assembly	Paxillin	TGFB1I1	Leupaxin
Saccharomyces cerevisiae	Budding Yeast	Fungi	R64-1-1	1	0	0
Caenorhabditis elegans	Roundworm	Invertebrate	WBcel235	1	0	0
Drosophila melanogaster	Fruitfly	Invertebrate	BDGP6	1	0	0
Ciona intestinalis	Sea Squirt	Urochordate	KH (GCA_000224145.1)	1	0	0
Petromyzon marinus	Sea Lamprey	Agnatha	Pmarinus_7.0	1	1	0
Danio rerio	Zebrafish	Teleostei	GRCz10	2	0	1
Oryzias latipes	Medaka	Teleostei	MEDAKA1	2	1	1
Oreochromis niloticus	Nile Tilapia	Teleostei	Orenil1.0	2	1	1
Poecilia formosa	Amazon Molly	Teleostei	PoeFor_5.1.2	2	1	1
Takifugu rubiripes	Fugu	Teleostei	FUGU4	2	1	1
Astyanax mexicanus	Mexican Cave Tetra	Teleostei	AstMex102	2	1	1
Xiphophorus maculatus	Platyfish	Teleostei	Xipmac4.4.2	2	1	1
Tetraodon nigroviridis	Tetraodon	Teleostei	TETRAODON 8.0	2	1	1
Lepisosteus oculatus	Spotted Gar	Holostei	LepOcu1	1	0	1
Callorhinchus milii	Elephant Shark	Holocephali	AAVX02000000	1	1	0
Latimeria chalumnae	Coelacanth	Sarcopterygii	LatCha1	1	1	1
Xenopus tropicalis	Western clawed frog	Amphibian	JGI_4.2	1	1	0
Anolis carolinensis	Anole lizard	Reptile	AnoCar2.0	1	1	1
Ficedula albicollis	Flycatcher	Avian	FicAlb_1.4	1	0	1
Gallus gallus	Chicken	Avian	Galgal4	1	0	1
Homo sapiens	Human	Mammal	GRCh38.p3	1	1	1
Mus musculus	Mouse	Mammal	GRCm38.p4	1	1	1

Table listing Paxillin protein family members identified through Ensembl database search.

**Fig 4:**
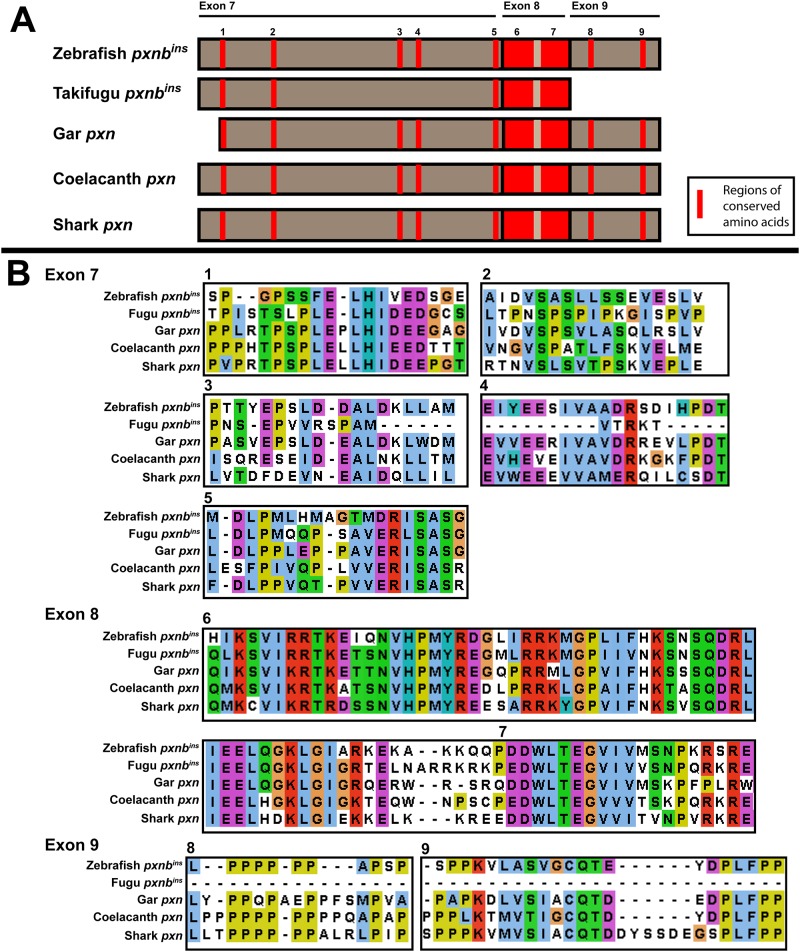
Conservation of *pxnb-ins* in non-Teleost fish. (**A**) Schematic diagram depicting exons 7–9 of zebrafish *pxnb-ins* compared with homologous regions in other species. Red bars represent regions of conservation between species. (**B**) Protein alignment of zebrafish Pxnb-ins and regions in other species colored based on conservation.

Identification of Paxillin family genes in more ancient fish further delineated the evolutionary history of these proteins. The Holosteian fish, represented by the spotted gar, did not undergo the same whole-genome duplication as Teleost fish. Accordingly, only one *pxn* gene and one *lpxn* gene were identified in this lineage. Surprisingly, no *tgfb1i1* gene was identified in the spotted gar genome, similar to the case in zebrafish. One of each Paxillin family member was identified in the coelacanth, a Sarcopterygian fish. Interestingly, both the coelacanth and spotted gar single *pxn* genes possess additional amino acids between their LD4 and LD5 motifs, partially homologous to the same region of the *pxnb-ins* isoform (Fig 4) found in zebrafish and other Teleosts. This observation suggests that an ancestral *pxn* gene contained this region and that it was lost in the Tetrapod lineage. Further support of this evolutionary model came from identification of a *pxn* gene in a representative cartilaginous fish, the elephant shark. Although only a partial genome assembly exists for this species, a single *pxn* gene was identified through our BLAST search. This *pxn* gene also had regions homologous to the amino acid insert region of the *pxnb-ins* isoform (Fig 4). Interestingly, however, the *pxn* genes of invertebrate and urochordate species examined did not have any homology to the amino acid insert region of *pxnb-ins*. These observations suggest that the extended region of amino acids between the LD4 and LD5 motifs of *pxn* arose shortly after the emergence of craniate animals and was subsequently lost during Tetrapod divergence from Sarcopterygian fish and in *pxna* genes of Teleost fish. Altogether, this phylogenetic analysis of *pxn* gene evolution revealed that the ancestral vertebrate *pxn* gene was likely most similar to zebrafish *pxnb*, including the *pxnb-ins* isoform, and that Tetrapod *pxn* genes and Teleost *pxna* genes emerged more recently.

TGFβ1I1 and Leupaxin genes were found to derive from a common ancestor, but had separate branches that were mostly consistent with species-level phylogenetic relationships. However, *Ciona* Paxillin was found to cluster with Leupaxin genes of other species. Relationships among Teleost Leupaxin and TGFβ1I1 orthologs are more consistent with the evolution of species in this lineage.

### Conserved synteny of Paxillin genes

**Fig 5:**
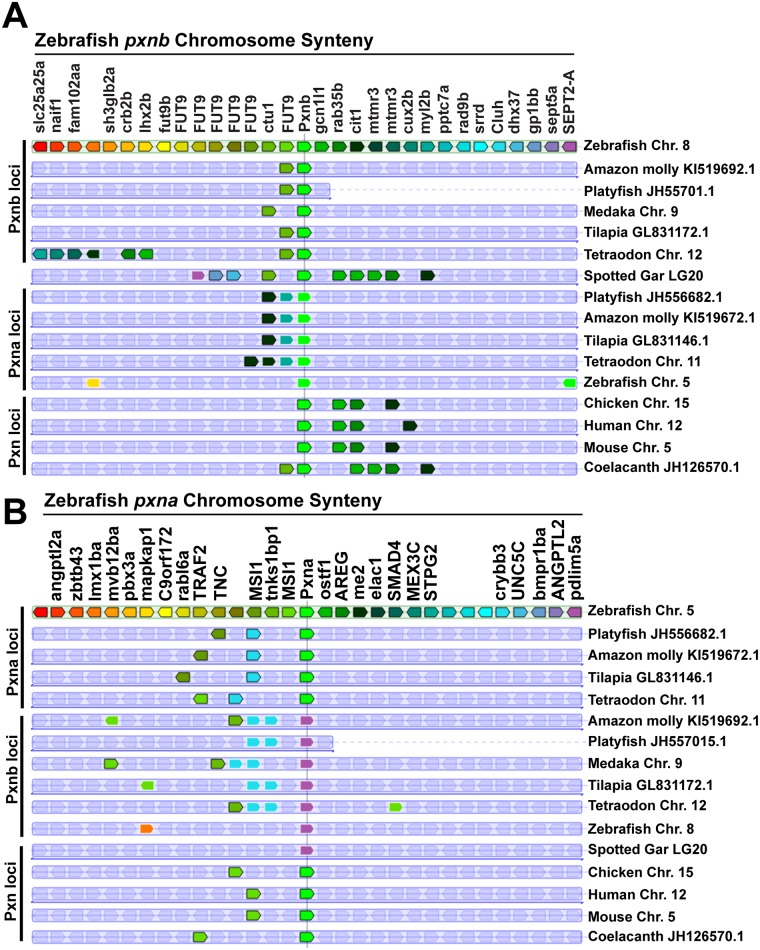
Synteny of Paxillin orthologs. (**A**) Local genomic arrangement around zebrafish *pxnb* in comparison with other *Paxillin* genes. (**B**) Local genomic arrangement around zebrafish *pxna* in comparison with with other *Paxillin* genes.

Analysis of chromosomal synteny between *pxn* genes across multiple taxa supported the hypothesis that the zebrafish *pxnb* gene is more similar to the vertebrate ancestral *pxn* gene prior to the Teleost-specific genome duplication than the *pxna* gene. Zebrafish *pxnb* is located on chromosome 8 along with the genes *ctu1*, *gcn1l1*, *rab35b* and *cit1* (Fig 5A). A similar genomic arrangement is observed in both the non-Teleost spotted gar and coelacanth, which retain an ancestral chromosomal landscape. Synteny between zebrafish *pxnb* and Tetrapod *pxn* genes was also observed, with all Tetrapod Paxillin genes being neighbored by both *gcn1l1* and *rab35* orthologs. Interestingly however, other Teleost *pxnb* genes were only adjacent to *ctu1*, while *cit1* orthologs in these fish were found on the same chromosome as their *pxna* orthologs. A similar syntenic relationship was found when *myl2b* was investigated. While in zebrafish *myl2b* was found on the same chromosome as *pxnb*, other Teleost *myl2* orthologs were found adjacent to *pxna*. These arrangements also support the hypothesis that Teleost *pxna* and *pxnb* genes arose from the duplication of an ancestral chromosome harboring *pxn*.

Synteny between the zebrafish *pxna* gene and other species was also investigated (Fig 5B). In zebrafish, *pxna* is found on chromosome 5 along with the genes *msi1* and *crybb3*. Orthologs of these genes were found on the same chromosome as *pxna* genes in all Teleosts investigated. In addition, coelacanth and Tetrapod *pxn* genes were also found on the same chromosome as their *msi1* orthologs. This limited syntenic relationship suggests that although the two *pxn* genes in Teleosts arose from a duplication event, further genomic rearrangements caused significant divergence from the ancestral *pxn*-containing chromosome around Teleost *pxna*. Furthermore, *crybb3* orthologs were found adjacent to both *pxna* and *pxnb* in all Teleosts except the zebrafish, suggesting that further significant rearrangements occurred after the Teleost-specific genome duplication on zebrafish chromosome 5.

Conservation of chromosomal synteny between *pxna* and *pxnb* reinforced the phylogenetic evidence that these genes are the product of a duplication event. Although the only paralogous genes other than *pxna* and *pxnb* retained locally on both zebrafish chromosomes 5 and 8 are *lmx1ba* and *lhx2b*, evidence of *pxn* chromosome duplication in Teleosts is stronger when other species are considered. The paralogous genes *crybb2* and *crybb3* are located adjacent to both *pxna* and *pxnb* in Nile tilapia, Tetraodon, Amazon molly, and platyfish. Additionally, *myl2* genes found neighboring zebrafish *pxnb* were also found neighboring *pxna* in other Teleosts. In Tetrapods, the single Paxillin gene was adjacent to genes orthologous to those found near both Teleost *pxna* and *pxnb*. Together, these findings suggest that a Paxillin-containing chromosome in an ancestral vertebrate was duplicated in the Teleost lineage to give rise to a new Paxillin ortholog.

### Spatial and temporal expression of Paxillin-a and Paxillin-b

**Fig 6:**
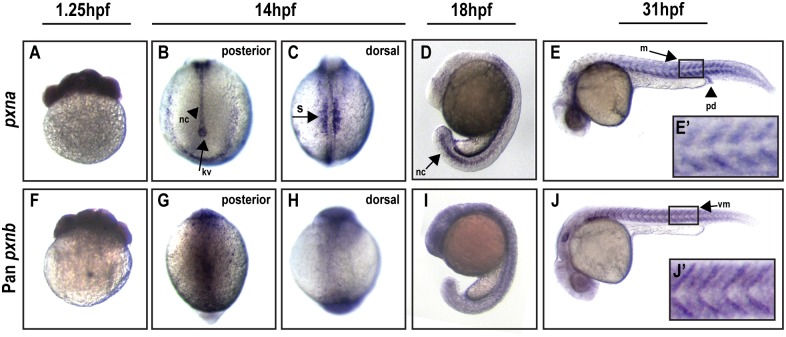
Spatial expression of Paxillin orthologs during early zebrafish development. (**A-E**) RNA *in situ* hybridizations using a *pxna* antisense probe detected maternal transcripts at 1.25 hpf (A) and enriched expression in posterior notochord (nc), Kupffer’s vesicle (kv), and somites (s) at 14 and 18 hpf (B-D). At 31 hpf (E), *pxna* expression was detected in pronephric ducts (pd) and myotomes (m). E’ depicts zoomed-in boxed regions of E. (**F-J**) A pan-*pxnb* antisense probe detected maternal mRNA (F) and unrestricted expression at 14 and 18 hpf (G-I). *pxnb* expression was detected in myotomes and vertical myosepta (vm) at 31 hpf (J). J’ depicts zoomed-in boxed regions of J.

The spatial expression patterns of *pxna* and *pxnb* transcripts were investigated in the zebrafish embryo via whole-mount RNA *in situ* hybridizations. We generated a *pxna*-specific probe, a pan-*pxnb* probe that detects all annotated *pxnb* isoforms and a *pxnb-ins*-specific probe (see Fig 2B). Each of these probes was used separately to characterize expression profiles of *paxillin* genes during zebrafish embryogenesis. This analysis indicated both *pxna* and *pxnb* transcripts are maternally deposited (Fig 6A and 6F) and ubiquitously distributed during gastrulation stages, which is consistent with *pxn in situ* hybridizations reported previously [18]. However, we observed unique tissue-restricted expression of different *pxn* transcripts during somitogenesis stages. *pxna* expression was enriched in developing somites and posterior notochord at 14 hpf (Fig 6B, 6C, 6G and 6H). Additionally, expression of *pxna* was observed in Kupffer’s vesicle (Fig 6B), a ciliated epithelial organ that orients the left-right body axis [28–30] During these stages of development, an ECM rich in Fibronectin and Laminin is generated around the notochord, somites, and Kupffer’s vesicle [31–33]. Previous antibody labeling has suggested that zebrafish Paxillin proteins may be crucial for adhesion and morphogenesis of these tissues at this stage [18]. At 18 hpf, during late somitogenesis, *pxna* transcripts remained enriched in the posterior notochord (Fig 6D). In contrast, pan-*pxnb* (Fig 6G–6I) or *pxnb-ins*-specific probes revealed unrestricted tissue expression of *pxnb* transcripts at 14 hpf and 18 hpf. At 31 hpf, *pxna* transcripts were enriched in the pronephric duct and developing myotomes (Fig 6E). At the same stage, *pxnb* transcripts were also enriched in the developing myotome (Fig 6J). However, unlike the broad *pxna* distribution in myoblasts between the vertical myoseptal regions (Fig 6E’), *pxnb* transcripts were localized at the vertical myosepta between somites (Fig 6J’). A similar myoseptal localization has been reported for other Integrin-adhesion complex mRNAs in the zebrafish embryo [34–36]. In addition, Dystroglycan complex proteins, also involved in cell-ECM adhesion, show transcript enrichment at the vertical myosepta similar to *pxnb* [37]. Intriguingly, local accumulation and translation of myosin mRNA has been demonstrated at sites of myoblast adhesion in culture in response to contractile forces [38]. Thus, *pxnb* mRNA distribution in the zebrafish myotome to vertical myosepta may be the result of muscle contraction and increased tension at this adhesion site. The functional relevance of polarized mRNA distribution in zebrafish embryonic muscle has not been investigated, although local translation at Integrin adhesion complexes has been suggested to be required for maintaining front-rear polarity and rapid downstream signaling in migrating cells [39, 40].

**Fig 7:**
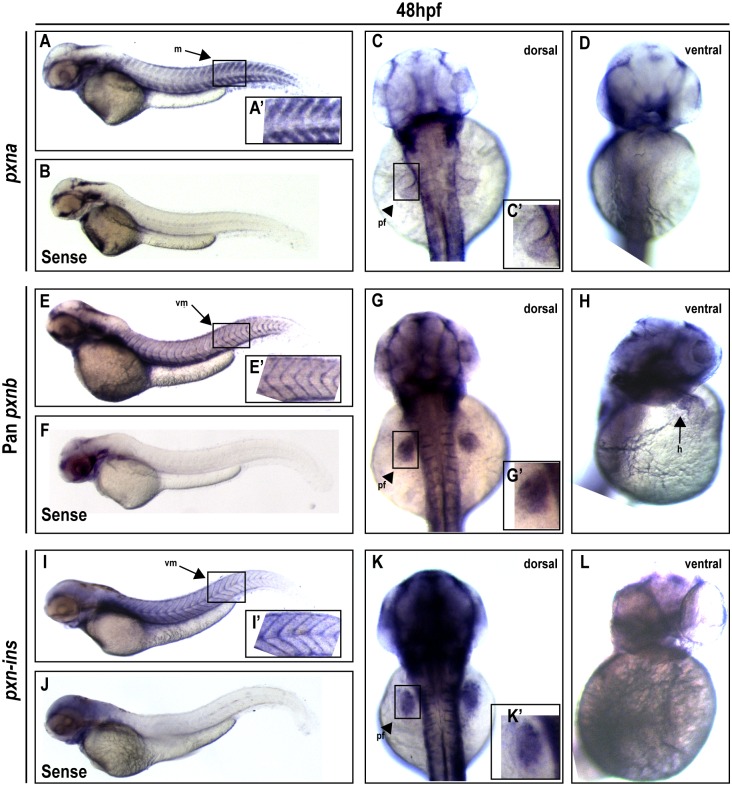
Expression profiles for Paxillin orthologs in the zebrafish embryo two days post-fertilization. (**A-D**) At 48 hpf, a *pxna* antisense probe detected specific expression in myotomes (m) (A) and the pectoral fin (pf) bud (C; dorsal view), when compared to background staining in the head revealed by control *pxna* (B), *pxnb* (F) and *pxnb-ins* (J) sense probes. A’ and C’ depict zoomed-in boxed regions in A and C. No *pxna* expression was observed in the heart (D; ventral view). (**E-H**) A pan-*pxnb* probe detected expression in vertical myosepta (vm) (E), pectoral fin bud (G) and heart (h) (H). E’ and G’ depict zoomed-in boxed regions of E and G. (**I-L**) A *pxnb-ins*-specific antisense probe detected expression in vertical myosepta (I) and pectoral fin bud (K), but no expression in the heart (L). I’ and K’ depict zoomed-in boxed region of I and K.

At 48 hpf, the complementary mRNA distribution of *pxna* and *pxnb* within the myotomes is maintained (Fig 7A, 7E, 7I and 7M) and expression of both *pxn* genes becomes enriched in other tissues. *pxna* and *pxnb* were detected in specific regions of the developing pectoral fin bud (Fig 7C, 7G and 7K). Other genes involved in ECM adhesion such as zebrafish Vinculin-b, α5-Integrin, and αV-Integrin are also expressed in this tissue at this stage [41–43]. These Integrin-adhesion complex proteins are likely required for adhesion to the ECM in this tissue [44], but may also be involved in growth factor signaling which regulates limb outgrowth [45–47]. Differential distribution of *pxna* and *pxnb* transcripts in the fin bud is suggestive of sub-functionalization for these genes in the development of this structure. While the transcript for *pxnb* and *pxnb-ins* are enriched in the fin bud mesenchyme[42], *pxna* transcript is more enriched in the apical ectodermal ridge [43, 48] cells involved in growth factor signaling to underlying fin bud mesenchymal cells [44]. The pan-*pxnb* probe detected expression in the embryonic heart at this stage (Fig 7D and 7H), but neither the *pxnb-ins* isoform nor *pxna* mRNA were detected in the heart (Fig 7L). These expression patterns suggest that each zebrafish Paxillin paralog and their splice isoforms play redundant and/or complementary roles in some developing tissues, and have gained uniquely regulated expression mechanisms for other tissues. Characterization of *pxna* and *pxnb* mRNA tissue distribution in embryos of other Teleost species will help elucidate conserved elements involved in spatial Paxillin gene expression.

**Fig 8:**
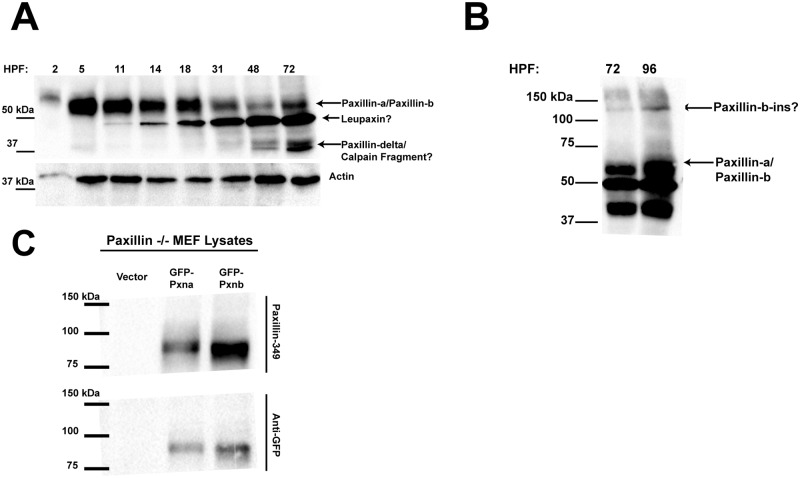
Temporal expression of Paxillin orthologs during zebrafish development. (**A**) Western blot analysis of wild-type zebrafish embryo lysates at the indicated hours post-fertilization (hpf) using Paxillin-349 antibody to detect Paxillin proteins and Actin antibody as a loading control. Bands corresponding to Pxna and Pxnb are indicated, and bands that potentially correspond to Leupaxin and Pxn-delta are pointed out. **B**) Western blot of lysates from late-stage wild type embryos. A band with the predicted molecular weight of Paxillin-b-ins is detected using the Paxillin-349 antibody. **C**) Paxillin-349 antibody Western blotting using lysates from Paxillin-null MEFs transfected with GFP-Pxna or GFP-Pxnb fusion proteins. GFP antibody was used to confirm intact fusion protein expression.

To address the expression of Paxillin proteins during development, Western blotting was performed on a temporal series of zebrafish embryo lysates (Fig 8A) using an existing antibody, Paxillin-349, which detects Paxillin as well as TGFβ1I1 in mammalian cells [16]. At 2 hpf, before zygotic transcription is initiated [49], a protein of roughly 60 kDa was detected. Computational predictions indicate that Pxna (58 kDa) and Pxnb (Zv9 = 63 kDa) proteins are consistent with this size. The ~60 kDa band is detected throughout development, while a second band of slightly less than 50 kDa is detected after gastrulation stages. The expression of this band is coincident with notochord and somite formation [50], and persists with increasing expression throughout the stages examined. Notably, the ~60 kDa band detected by this antibody has decreased expression relative to the ~50 kDa band from 31 hpf to 72 hpf. Although the 50 kDa band is a similar molecular weight to mammalian TGFβ1I1, no TGFβ1I1 gene was annotated in the zebrafish genome. However, the zebrafish Leupaxin-like protein and mammalian TGFβ1I1 share strong amino-acid homology within LD motifs, and is predicted to be 44 kDa which is consistent with the size of the protein detected. A third band of ~40 kDa is detected at 48 hpf that persists through 72 hpf. Intriguingly, a short Paxillin protein isoform, called Paxillin delta [51], is generated from an internal ribosome entry sequence on the full-length Paxillin transcript. Expression of this isoform has been noted to be upregulated in mammalian epithelial cell types. Multiple nucleotide alignment shows that this internal ribosome entry sequence is conserved in many vertebrate species, including zebrafish [51]. However, it remains to be determined if the lowest molecular weight band detected from the embryonic lysate is this isoform, since cleavage of full-length Paxillin by Calpain has also been reported to generate a peptide with a similar size to Paxillin delta [52]. Although the *pxnb-ins* transcript was detected at all stages of embryonic development by RT-PCR (Fig 2C), a protein band of the predicted molecular weight (130 kDa) of this isoform was not detected during early embryogenesis. Interestingly, however, a band of this size was detected at relatively low levels in lysates from later staged embryos (Fig 8B) suggesting that this isoform’s expression may be regulated post-transcriptionally.

To determine whether the Paxillin-349 antibody detected zebrafish Pxna, Pxnb, or both, we generated GFP fusion proteins. Lysates from mammalian cells overexpressing either GFP-Pxna or GFP-Pxnb were used to further characterize the specificity of the Paxillin-349 antibody. In this context, both fusion proteins were detected (Fig 8C), indicating the antibody can recognize both Pxna and Pxnb. These results validate the cross-reactivity of Paxillin-349 antibody with zebrafish Paxillin proteins and highlight the need to develop specific antibodies to further characterize the expression profiles of zebrafish Pxna and Pxnb proteins.

### Conservation of zebrafish Paxillin protein localization in Mammalian cells

**Fig 9:**
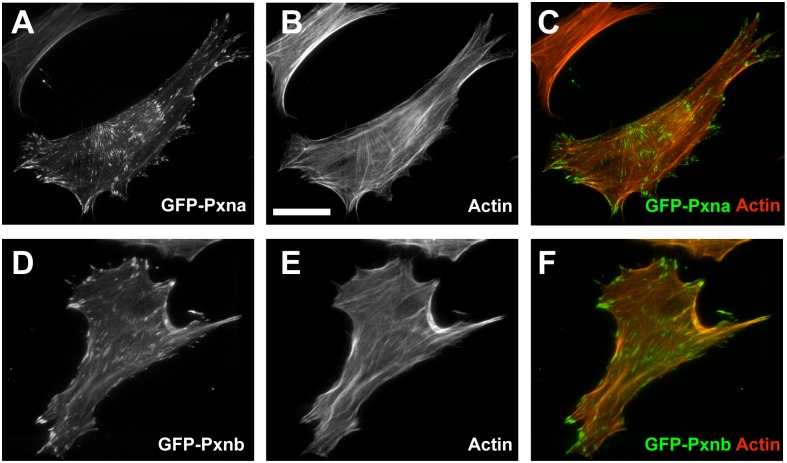
Subcellular localization of zebrafish Pxna and Pxnb proteins in mammalian cells. Paxillin-null mouse embryonic fibroblasts transfected with GFP-Pxna (**A-C**) or GFP-Pxnb (**D-F**). Both GFP-Pxna (A) and GFP-Pxnb (D) fusion proteins localize to focal adhesions similar to mammalian Paxillin proteins. Phalloidin was used to counter-stain f-Actin (B,E). Merged channels are shown in (C,F). Scale bar = 5μm.

In order to examine potentially evolutionarily conserved functions of zebrafish Paxillin family members, N-terminal GFP-fusion proteins were expressed mammalian cells. Endogenous Paxillin protein localizes to focal adhesions at the tips of actin stress fibers [12]. Expression of either zebrafish GFP-Pxna or GFP-Pxnb in Paxillin-null mouse embryonic fibroblasts (MEFs) [15] plated on Fibronectin also localized to focal adhesions (Fig 9). This subcellular distribution shows that both zebrafish Paxillin orthologs are receptive to mammalian adhesion signaling, and may act as regulators downstream of Integrin adhesion in their native environments. This result is consistent with the high amino acid conservation between LIM domains, required for focal adhesion localization [3], of these proteins. Since zebrafish Paxillin proteins can localize to Integrin adhesion sites in mammalian cells, and localize to myotendinous junctions endogenously, we predict these genes play analogous roles in Integrin adhesion at these structures.

Identifying and characterizing the expression of zebrafish *pxnb* is an essential first step to using zebrafish as a model system to elucidate functional roles for Paxillin family members during vertebrate development. In-depth analysis of the molecular evolution of this gene revealed that the ancestral vertebrate Paxillin gene likely produced splice isoforms similar to both Pxna and Pxnb, but Pxnb was lost in lineages other than Teleosts. Interestingly, these genes have overlapping and unique expression profiles in zebrafish embryos. Future studies are now possible to determine whether these Paxillin proteins serve compensatory, complementary, or antagonistic roles during embryogenesis.

## Experimental Procedures

### Zebrafish

The project described was reviewed and approved by SUNY Upstate Medical University's IACUC (#363). The University has an Animal Welfare Assurance on file with the Office of Laboratory Animal Welfare. The Assurance Number is A3514-01. The wild-type TAB strain of zebrafish (*Danio rerio*) was housed according to standard protocols [53]. Embryonic stage of offspring collected from natural matings was determined as described previously [50].

### Gene identification

The full-length human (*Homo sapiens*) Paxillin protein sequence (Ensembl ID: ENST00000424649) was used for a BLASTP search to identify Paxillin family members in the zebrafish genome (Ensembl Genome assembly Zv9). The full-length zebrafish Paxillin-b protein sequence (Ensembl ID: ENSDART00000085993) was then used for a BLASTP search to identify other Paxillin family member orthologs in the remaining genomes used for this study in Ensembl and NCBI databases (Table 1).

### Phylogenetic analysis

Full-length Paxillin family protein sequences from selected species were aligned using T-Coffee [54]. The appropriate amino-acid substitution model to infer phylogeny was determined using Maximum Likelihood implemented in MEGA6 [55]. The resulting alignment was used to generate a Maximum Likelihood tree using the JTT+G+I substitution model and 1000 bootstrap iterations. Syntenic relationships between Paxillin family member genes across species were visualized using default settings with Genomicus v81.01 [56].

### RT-PCR

Total RNA from pooled zebrafish embryos of the same stage was extracted using QiaZOL (Qiagen) and precipitated using isopropanol. Extracted RNA (500ng/each stage) was used for cDNA synthesis with the iScript cDNA synthesis kit (BioRad). PCR was then performed using EconoTaq (Lucigen).

Primers used: *pxna* E7 forward: 5’-AGGAAATCGGCTCCAGAAAT-3’, *pxna* E10 reverse: 5’-GCCAGAAGGTGACGCTATTT-3’; *pxnb* E2 forward: 5’-CCAGTGGAGGTCAGCTGTTT-3’, *pxnb* E6 reverse: 5’-TCCTCAAGTTCTCGTGTGGC-3’, *pxnb-ins* E7 forward: 5’-CAGAGCTCTCTCACCTCC-3’, *pxnb-ins* E10 reverse: 5’-CACACTCCTTTGGCAACC-3’.

### Western blotting

Pooled zebrafish embryos of the same stage were manually dechorionated with forceps and deyolked as previously described [57]. Samples were then homogenized in 2x SDS-PAGE sample buffer using 2μL of buffer per embryo and boiled. Western blotting was performed using mouse anti-Paxillin (clone 349, BD Trans), mouse anti-Actin (Millipore), or mouse anti-GFP (Santa Cruz) primary antibodies at 1:1000 dilution, anti-mouse HRP secondary antibody (BioRad) was used at 1:10,000 dilution.

### In Situ hybridization

cDNA probes were generated by RT-PCR using primers:

*pxna* E2 forward: 5’-GAGTCCACAACCTCCCACAT-3’, *pxna* E6 reverse: 5’-ACCGTCCCGCTCAAAGAAAT-3’;

*pxnb* E2 forward: 5’-CCAGTGGAGGTCAGCTGTTT-3’, *pxnb* E6 reverse: 5’-TCCTCAAGTTCTCGTGTGGC-3’;

*pxnb* E7 forward: 5’-CAGAGCTCTCTCACCTCC-3’, *pxnb* E10 reverse: 5’-CACACTCCTTTGGCAACC-3’

*pxnb-ins* E7 forward: 5’-CAGAGCTCTCTCACCTCC-3’, *pxnb-ins* E10 reverse: 5’-CACACTCCTTTGGCAACC-3’

PCR products were cloned into the dual-promoter pCRII TOPO vector (Invitrogen). *In vitro* DIG-labeled sense and antisense RNA probe synthesis was performed using linearized plasmids and SP6 and T7 RNA polymerase *in vitro* transcription reagents (Roche). Whole zebrafish embryos were fixed in 4% paraformaldehyde/1X sucrose solution at 4°C overnight. *In situ* hybridization was performed as previously described [58] and detected using NBT/BCIP (Roche) as a chromogenic substrate.

### GFP-construct immunofluorescence

Full-length zebrafish *pxna* was amplified from cDNA using primers: *pxna* ORF forward: 5’-ATGGATGACCTTGACGCATTATTGGCGGATTTGGAGTCC-3’, *pxna* ORF rvr: 5’-GCCACAAGGTGACGCTATTTGCTC-3’; partial zebrafish *pxnb* was generated using *pxnb* Zv9 E1 forward: 5’-ATGACTTACGTCTACTGTGTGTTCCTCCC-3’ and *pxnb* E6 reverse: 5’-TCCTCAAGTTCTCGTGTGGC-3’ or *pxnb* E2 forward: 5’-CCAGTGGAGGTCAGCTGTTT-3’ and *pxnb* ORF reverse: 5’-GCACACGTCTAGCTGAAGAGCTTG-3’. These partial transcripts were ligated together with an internal BssSI restriction enzyme site in *pxnb* exon 6. Primers engineered with restriction enzyme sites at the 5’ and 3’ ends of each cDNA were designed for generating GFP-fusion constructs in pCS2+ plasmids. Paxillin-null MEF cells cultured with DMEM were transfected with GFP-zebrafish Pxna or GFP-zebrafish Pxnb in pCS2+ plasmids using TransIT-X2 (Mirus). Cells were then allowed to spread for 24 hours on Fibronectin-coated coverslips. Cells were fixed and permeabilized with 4% paraformaldehyde/0.1% TritonX-100, quenched with 0.1M glycine, then blocked overnight at 4°C in 3% BSA/PBS. Rhodamine Phalloidin was then incubated at 1:1000 dilution in PBS+0.05% Tween-20. Coverslips were mounted on slides using Gelvatol and imaged using a Carl Zeiss compound microscope.
